# *Basidioradulum mayi* and *B. tasmanicum* spp. nov. (*Hymenochaetales*, *Basidiomycota*) from both sides of Bass Strait, Australia

**DOI:** 10.1038/s41598-019-57061-y

**Published:** 2020-01-09

**Authors:** Xue-Wei Wang, Ji-Hang Jiang, Li-Wei Zhou

**Affiliations:** 1 Institute of Applied Ecology, Chinese Academy of Sciences, Shenyang, 110016 P.R. China; 2 State Key Laboratory of Mycology, Institute of Microbiology, Chinese Academy of Sciences, Beijing, 100101 P.R. China; 3 University of Chinese Academy of Sciences, Beijing, 100049 P.R. China

**Keywords:** Fungal evolution, Fungal ecology

## Abstract

*Basidioradulum* was morphologically considered to be a synonym of *Xylodon*. Here, its independence within *Hymenochaetales* is confirmed from a phylogenetic perspective. *Basidioradulum radula*, the generic type, is widely distributed in Northern Hemisphere. Two Southern Hemisphere species close to *B. radula* are newly described as *B. mayi* and *B. tasmanicum*, respectively, from Victoria and Tasmania, Australia. *Basidioradulum mayi* differs from *B. radula* by lack of cystidia. Moreover, the hymenial surface of *B. radula* is normally much more strongly hydnoid than that of *B. mayi*. *Basidioradulum tasmanicum* is distinct from *B. radula* and *B. mayi* by having capitate cystidia, ellipsoid to subglobose basidiospores, and crystal-covered hyphae. Although morphologically distinct, the two new species isolated by Bass Strait have an almost identical ITS region, and could not be differentiated by nLSU- and ITS-based phylogenetic analyses. This case reminds us that basing phylogeny simply on the ITS as a barcode region may underestimate fungal species diversity.

## Introduction

*Basidioradulum* Nobles was introduced for *B. radula* (Fr.) Nobles^1^. The generic type of the genus, *B. radula*, was combined as *Xylodon radula* (Fr.) Ţura, Zmitr., Wasser & Spirin based on morphological characters^2^. However, phylogenetic studies including this species all failed to group *X. radula* with other species of *Xylodon* (Pers.) Gray^3–5^. Meanwhile, morphologically fungal taxonomists have not widely accepted this species as a member of *Xylodon* or even of *Schizoporaceae*^6,7^. A further 10 species have been placed at some time in *Basidioradulum*. According to Index Fungorum (http://www.indexfungorum.org/), nine of these belong elsewhere. The remaining species, *B. crustosum* (Pers.) Zmitr., Malysheva & Spirin is an invalid combination in *Basidioradulum* according to the Arts. 41.5, 41.8(a), and 41.8(b) of the International Code of Nomenclature^8^.

During an examination of wood-inhabiting fungi from Australia, our attention was drawn to the relationships of five specimens with similarities to *Basidioradulum radula*. After taking Chinese specimens of *B. radula* into consideration together, we described the Australian specimens with almost identical ITS sequences as two new species based on extremely distinct morphological characters and localities separated by Bass Strait.

## Results

**Figure 1:**
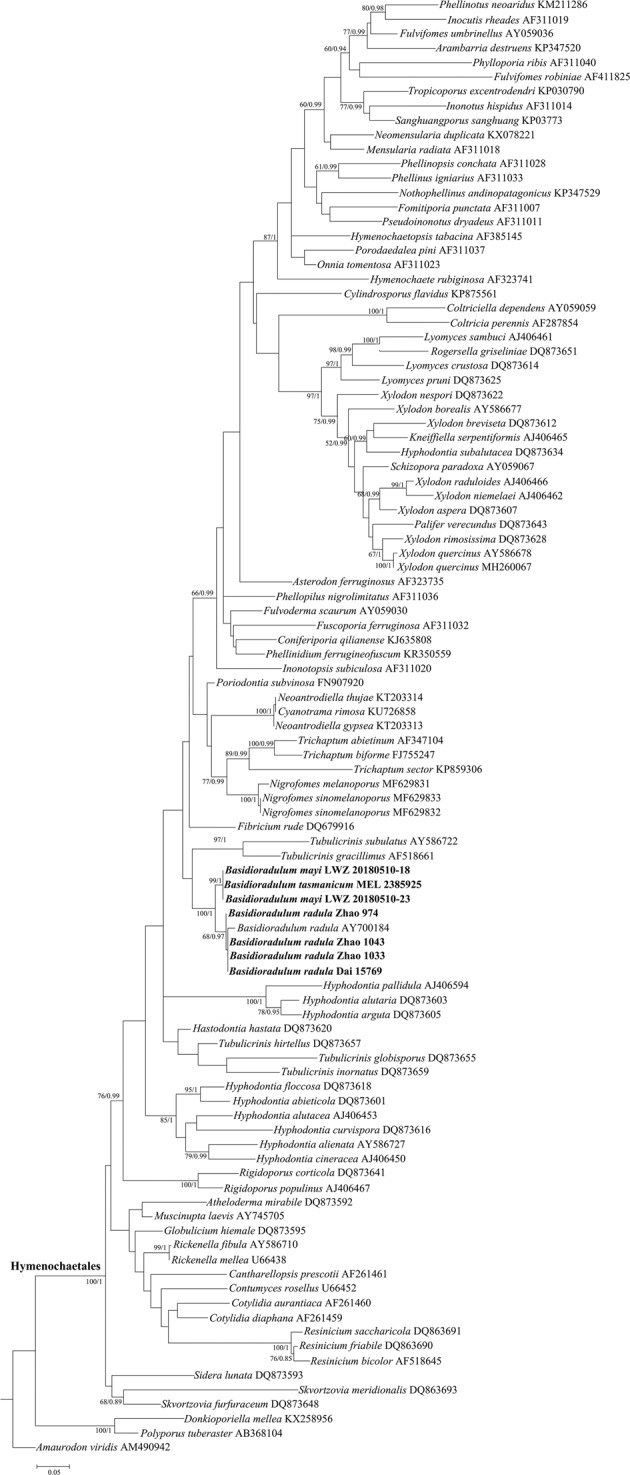
Phylogenetic position of *Basidioradulum* inferred from the nLSU regions. The topology generated from the maximum likelihood method is presented. Values at nodes are bootstrap and Bayesian posterior probability, respectively, above 50% and 0.8. Newly sequenced specimens are in bold.

**Figure 2:**
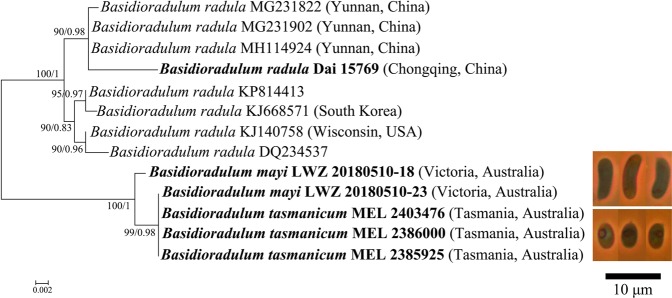
Phylogenetic relationship among species of *Basidioradulum* inferred from the ITS region. The topology generated from the maximum likelihood method is presented. Values at nodes are bootstrap and Bayesian posterior probability, respectively, above 50% and 0.8. Newly sequenced specimens are in bold. The voucher locations of sequences are labeled in parentheses if available. The photos of basidiospores of *B. mayi* and *B. tasmanicum* taken from the holotypes are presented after their species names.

Nine specimens from China and Australia were sequenced for this study, which resulted in seven new nLSU sequences and six new ITS sequences (Table 1). For the nLSU alignment of 101 taxa with 929 characters, the ML search stopped after 400 BS replicates, and all chains converged indicated by the effective sample sizes (ESSs) of all parameters above 2500 and the potential scale reduction factors (PSRFs) equal to 1000. With regard to the ITS alignment of 13 taxa with 573 characters, after 300 BS replicates the ML search stopped, while the ESSs of all parameters above 6000 and the PSRFs equal to 1000 suggested the convergence of all chains. The two phylogenetic methods generated nearly congruent topologies for both nLSU and ITS alignments. Therefore, the topologies generated from the ML method are presented along with the statistical values at the nodes of BS and BPPs above 50% and 0.8, respectively (Figs. 1 and 2).

**Table 1:** Information on specimens newly sequenced for this study.

Species	Specimen number	GenBank accession number		Origin
		nLSU	ITS	
*B. radula*	Zhao 1043 (SWFC)	MN017787	MG231822^a^	Yunnan, China
*B. radula*	Zhao 1033 (SWFC)	MN017788	MG231902^a^	Yunnan, China
*B. radula*	Zhao 974 (SWFC)	MN017789	MH114924^a^	Yunnan, China
*B. radula*	Dai 15769 (BJFC)	MN017790	MN017786	Chongqing, China
*B. mayi*	LWZ 20180510-18 (IFP)	MN017792	MN017785	Victoria, Australia
*B. mayi*	LWZ 20180510-23 (IFP)	MN017791	MN017784	Victoria, Australia
*B. tasmanicum*	MEL 2385925	MN017793	MN017783	Tasmania, Australia
*B. tasmanicum*	MEL 2403476	—	MN017781	Tasmania, Australia
*B. tasmanicum*	MEL 2386000	—	MN017782	Tasmania, Australia

^a^Downloaded from GenBank.

The phylogeny inferred from the nLSU region did not classify families well within *Hymenochaetales* (Fig. 1), but did separate the fully supported *Basidioradulum* lineage from other species belonging to *Schizoporaceae*, including *Xylodon quercinus* (Pers.) Gray, the generic type of *Xylodon*. The *Basidioradulum* lineage was divided into two clades. The clade of *B. radula* with five Northern Hemisphere specimens received a moderate BS value of 68% and a high BPP value of 0.97. The Southern Hemisphere clade with three Australian specimens was strongly supported (99% BS, 1 BPP).

The midpoint-rooted tree inferred from the ITS region indicates that all 13 collections of *Basidioradulum* formed two fully supported clades (Fig. 2). One clade including eight collections originating from the Northern Hemisphere represented *B. radula*, while the other clade had five Southern Hemisphere collections.

The pairwise distance matrix for ITS sequences of *Basidioradulum* (Table 2) shows that the 13 collections were separated into two groups that are congruent with the two clades in the ITS-based phylogeny (Fig. 2). The distances among Group 1 including five Southern Hemisphere collections and among Group 2 including eight Northern Hemisphere collections were, respectively, 0.2% and 0.9%, while the distance between the two groups was 3.4%.

**Table 2:** Matrix of pairwise differences among ITS sequences from collections of three species of *Basidioradulum*.

Species	1	2	3	4	5	6	7	8	9	10	11	12	13
**Group 1**													
1. *B. tasmanicum* (MEL 2403476)													
2. *B. tasmanicum* (MEL 2386000)	0.000												
3. *B. tasmanicum* (MEL 2385925)	0.000	0.000											
4. *B. mayi* (LWZ 20180510-23, IFP)	0.000	0.000	0.000										
5. *B. mayi* (LWZ 20180510-18, IFP)	0.005	0.005	0.005	0.005									
**Group 2**													
6. *B. radula* (MH114924)	0.032	0.032	0.032	0.032	0.031								
7. *B. radula* (MG231902)	0.032	0.032	0.032	0.032	0.031	0.000							
8. *B. radula* (KJ668571)	0.036	0.036	0.036	0.036	0.034	0.009	0.009						
9. *B. radula* (MG231822)	0.034	0.034	0.034	0.034	0.032	0.002	0.002	0.011					
10. *B. radula* (KJ140758)	0.034	0.034	0.034	0.034	0.032	0.007	0.007	0.005	0.009				
11*. B. radula* (Dai 15769, BJFC)	0.038	0.038	0.038	0.038	0.036	0.011	0.011	0.020	0.013	0.018			
12*. B. radula* (DQ234537)	0.035	0.035	0.035	0.035	0.033	0.011	0.011	0.009	0.013	0.004	0.014		
13*. B. radula* (KP814413)	0.034	0.034	0.034	0.034	0.032	0.005	0.005	0.002	0.007	0.004	0.016	0.007	

All the above molecular evidence supported the Southern Hemisphere specimens as distinct from *Basidioradulum radula*. Moreover, further morphological examinations indicated that among the Southern Hemisphere specimens the two Victorian specimens were extremely different to the three Tasmanian specimens. Therefore, two new species of *Basidioradulum* are described below.

## Taxonomy

**Figure 3:**
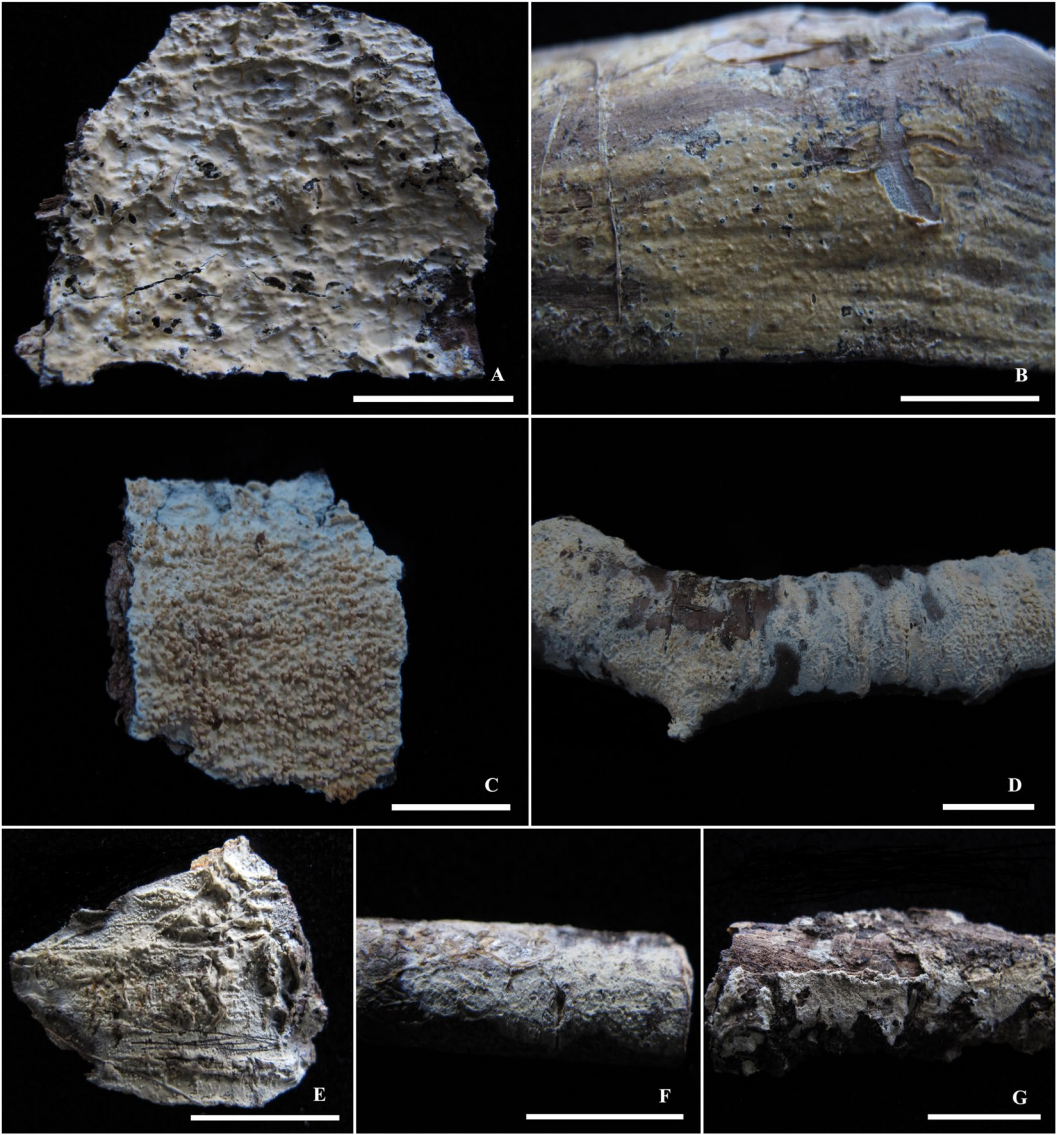
Basidiocarps of *Basidioradulum mayi* and *B. tasmanicum*. (**A**) *B. mayi* (LWZ 20180510-23, holotype). (**B**) *B. mayi* (LWZ 20180510-18). (**C**) *B. radula* (Zhao 1043). (**D**) *B. radula* (Dai 15769). (**E**) *B. tasmanicum* (MEL 2403476). (**F**) *B tasmanicum* (MEL 2386000). (**G**) *B. tasmanicum* (MEL 2385925, holotype). Scale bars = 1 cm.

**Figure 4:**
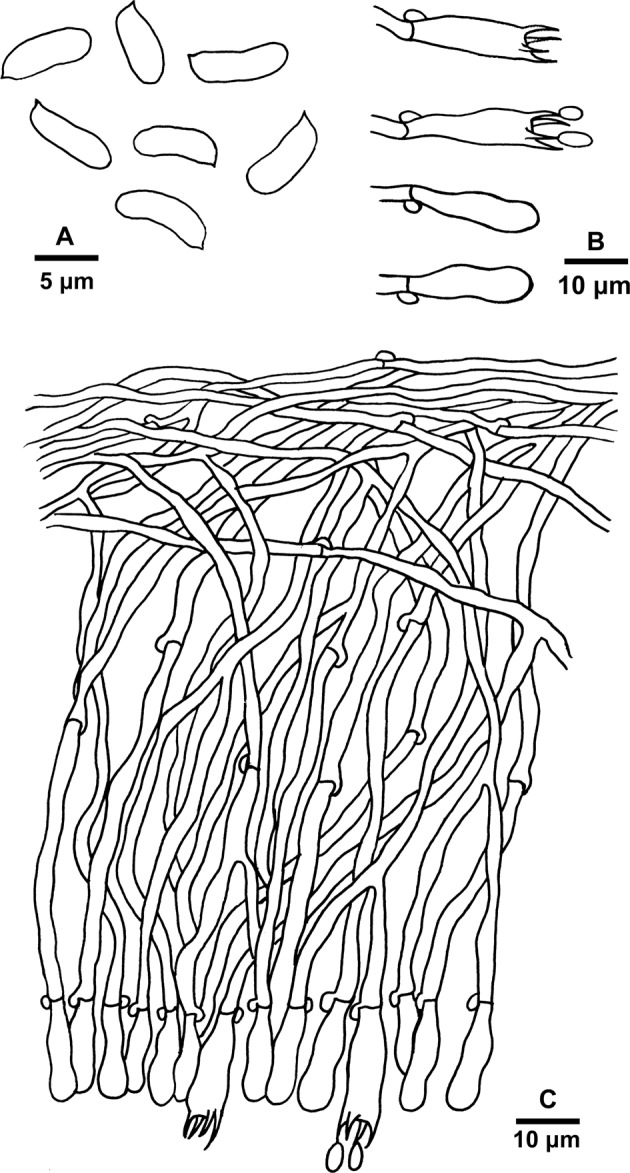
Microscopic structures of *Basidioradulum mayi* (drawn from the holotype). (**A**) Basidiospores. (**B**) Basidia and basidioles. (**C**) A section of the basidiocarp.

***Basidioradulum mayi*** Xue W. Wang & L.W. Zhou, sp. nov. Figs. 3 and 4

MycoBank: MB 833704.

Etymology: *mayi* (Latin), in honor of Australian mycologist, Dr. Tom May, who kindly arranged the author Li-Wei Zhou’s field trip in Victoria, Australia.

Type: AUSTRALIA: Victoria, Yarra Ranges National Park, Cora Lynn Falls, on the base of dead standing angiosperm, 10 May 2018, *LWZ 20180510-23* (holotype in MEL, isotype in IFP).

Basidiocarps annual, effused, adnate, hard and cracked when dry. Hymenial surface smooth, odontoid to hydnoid, the teeth conical or irregular, up to 1–2 mm long and 0.5–1 mm wide, becoming gradually shorter towards margin, whitish-cream to light-ochraceous. Margin slightly fibrillose, paler or concolorous with fertile area. Subiculum homogeneous, whitish-cream, up to 0.8 mm thick.

Hyphal system monomitic; generative hyphae with clamp connections. Subicular hyphae hyaline, thin- to slightly thick-walled, occasionally branched, more or less parallel to substrate, 2.5–4.5 μm wide. Subhymenial hyphae somewhat horizontal to vertical along substrate, hyaline, agglutinated, 2.5–4.5 μm wide. Cystidia absent. Basidia clavate to subclavate, 20–25 × 5.5–7.5 μm, with 4 sterigmata each 2–3 μm long and a clamp connection at the base. Basidioles similar in shape to basidia, but smaller. Basidiospores cylindrical to slightly allantoid, hyaline, smooth, thin-walled, CB−, IKI–, (7.2−)7.3–9.5(−9.7) × (2.1−)2.2–3.5(−3.7) μm, L = 8.20 μm, W = 2.92 μm, Q = 2.80–2.82 (60/2).

Additional specimen (paratype) examined: AUSTRALIA: Victoria, Yarra Ranges National Park, Cora Lynn Falls, on fallen angiosperm branch, 10 May 2018, *LWZ 20180510-18* (IFP, MEL).

Notes: The smooth, odontoid to hydnoid hymenial surface, clavate to subclavate basidia, and cylindrical to slightly allantoid basidiospores indicate that the new species is close to *Basidioradulum radula*. However, *B. mayi* differs by lack of cystidia and being found in Australia in the Southern Hemisphere. Moreover, the hymenial surface of *B. radula* as exemplified by Chinese specimens (Fig. 3) is normally much more strongly hydnoid than that of *B. mayi*.

**Figure 5:**
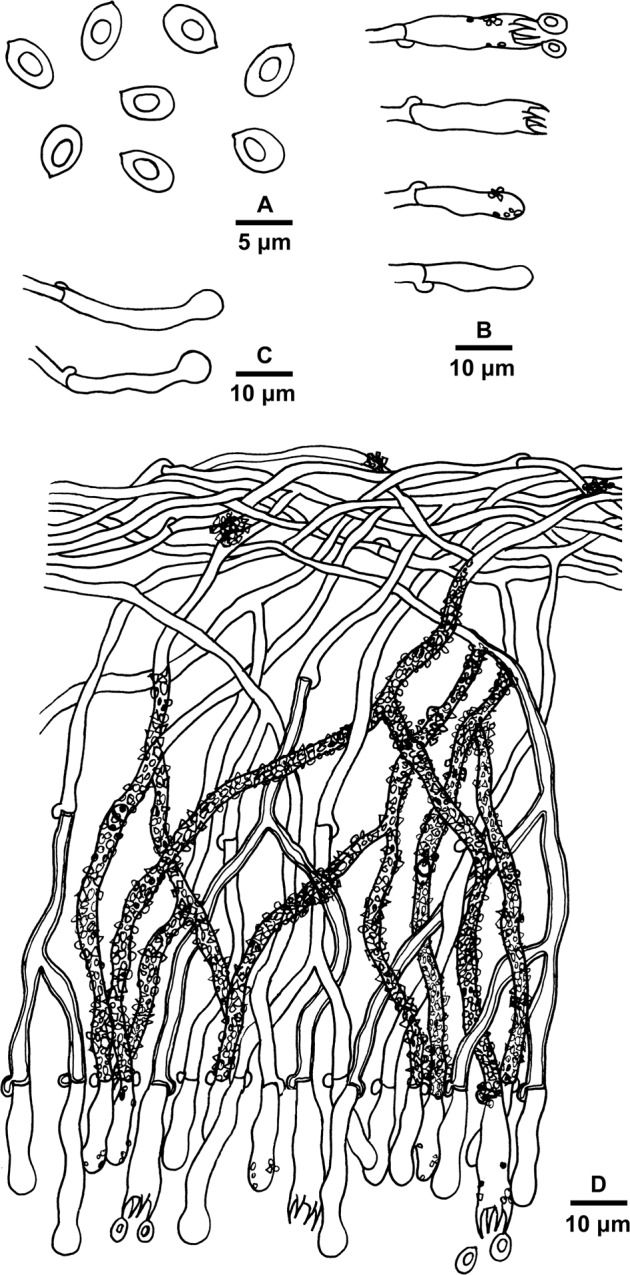
Microscopic structures of *Basidioradulum tasmanicum* (drawn from the holotype). (**A**) Basidiospores. (**B**) Basidia and basidioles. (**C**) Capitate cystidia. (**D**) A section of the basidiocarp.

***Basidioradulum tasmanicum*** Xue W. Wang & L.W. Zhou, sp. nov. Figs. 3 and 5.

MycoBank: MB 833705.

Etymology: *tasmanicum* (Latin), refers to the island of Tasmania, Australia.

Type: AUSTRALIA: Tasmania, Sandspit Forest, Wielangta Rainforest Reserve, on fallen angiosperm branch, 8 August 2006, *N. Hallenberg 15784* (holotype MEL 2385925).

Basidiocarps annual, effused, adnate, cracked when dry. Hymenial surface smooth or with various kinds of projections, cream to buff. Margin slightly pilose, paler or concolorous with fertile area. Subiculum homogeneous, whitish-cream.

Hyphal system monomitic; generative hyphae with clamp connections. Subicular hyphae hyaline, thin- to slightly thick-walled, occasionally branched, more or less parallel to substrate, 2.5–4.5 μm wide. Subhymenial hyphae somewhat horizontal to vertical along substrate, hyaline, agglutinated, usually covered with granular crystals, capitate terminal branches numerous, 2.5–4.5 μm wide. Capitate cystidia thin-walled, projecting for approximately half their lengths, 20–35 × 3.5–4.5 μm. Basidia clavate to subclavate, 20–25 × 5.5–7.5 μm, with 4 sterigmata each 2–3 μm long and a clamp connection at the base. Basidioles similar in shape to basidia, but smaller. Basidiospores ellipsoid or subglobose, with a large oil drop, hyaline, smooth, thin-walled, CB–, IKI−, (4.6–)4.7–5.8(−6.5) × (2.3−)2.4–3.7(−3.9) μm, L = 5.20 μm, W = 3.07 μm, Q = 1.60–1.80 (90/3).

Additional specimens (paratypes) examined: AUSTRALIA: Tasmania, Franklin-Gordon Wild Rivers National Park, Franklin River Nature Trail, on fallen angiosperm branch, 13 August 2006, *N. Hallenberg 15892* (MEL 2386000); Tasmania, Franklin-Gordon Wild Rivers National Park, Franklin River Nature Trail, on dead wood, 15 April 2008, *G.M. Gates & D.A. Ratkowsky FF328* (MEL 2403476).

Notes: *Basidioradulum tasmanicum* is distinct from *B. radula* and *B. mayi* by having capitate cystidia, ellipsoid to subglobose basidiospores, and crystal-covered hyphae.

## Discussion

In this study, the phylogenetic position of *Basidioradulum* was reevaluated by sampling more collections of this genus in the nLSU-based phylogeny (Fig. 1). Although the resolution at the family level was poor, *Basidioradulum* was clearly separated from *Xylodon quercinus*, the generic type of *Xylodon*, as in previous studies^3–5^. Therefore, we reject the transfer of *B. radula*, the generic type of *Basidioradulum* to *Xylodon* proposed by Ţura *et al*.^2^, and treat *Basidioradulum* as a distinct genus from *Xylodon*. Because comprehensive phylogenetic sampling of taxa of the order *Hymenochaetales* is lacking, we consider *Basidioradulum* to be a genus *incertae sedis* at the family rank within *Hymenochaetales*.

*Basidioradulum radula* as defined morphologically is considered to be widely distributed in Northern Hemisphere^2^. The sequences of *B. radula* from Chinese, Korean and American collections analyzed in this study also confirm its wide distribution. Meanwhile, the two Southern Hemisphere relatives of *B. radula* are distinct, and newly described as *B. mayi* and *B. tasmanicum*. These two species cannot be differentiated by molecular evidence on the basis of ITS region, which is used as the fungal barcode (Fig. 2, Table 2). The situation where *B. mayi* and *B. tasmanicum* have distinct morphological characters but cannot be delimited by DNA data indicates that these two species isolated by Bass Strait may be undergoing an ongoing allopatric speciation event^9^. However, more evidence, such as mating test between these two species and multi locus-based phylogeny, is needed to further confirm this event. Nevertheless, the current case of *Basidioradulum* does stimulate consideration of the multitude of species concepts and recognition criteria as applied to fungi^10,11^. Although the molecular phylogenetic method normally has been considered to be a powerful means to discover new fungal lineages^12,13^, it is concluded from the current case that if we only consider differences in the ITS region, fungal diversity may be underestimated. The speciation of fungi needs time, and during the process of speciation the divergence of phenotype and genotype could happen at differentiated paces. Therefore, neither the genotype nor phenotype alone is suitable to be used as evidence to describe species. Instead, polyphasic evidence from morphology, phylogeny, ecology and so on, if available, should be used together to delimit fungal species.

## Conclusion

Two wood-inhabiting basidiomycetous species *Basidioradulum mayi* and *B. tasmanicum* are newly described from both sides of Bass Strait, Australia. These two species have almost identical fungal barcoding ITS sequences but distinct morphological characters.

## Materials and Methods

The studied specimens are deposited at the herbaria of the Institute of Applied Ecology, Chinese Academy of Sciences (IFP), Shenyang, China, the Institute of Microbiology, Beijing Forestry University (BJFC), Beijing, China, Southwest Forestry University (SWFC), Kunming, China, and the National Herbarium of Victoria (MEL), Melbourne, Australia.

The hymenial surfaces of basidiocarps were observed under a stereomicroscope. Special color terms followed Petersen^14^. Microscopic characters were examined using a Nikon Eclipse 80i microscope at magnification up to 1000×. Specimen sections were stained in Cotton Blue (CB), Melzer’s reagent (IKI) and 5% potassium hydroxide. All measurements were taken from CB-stained sections. The basidiospore size variation was presented by putting 5% of measurements from each end of the range in parentheses. Drawings were made with the aid of a drawing tube. Photos of basidiospores were taken using a Nikon Digital Sight DS-U3 camera. The following abbreviations are used in the text: L = mean basidiospore length (arithmetic average of all basidiospores), W = mean basidiospore width (arithmetic average of all basidiospores), Q = variation in the L/W ratios between the specimens studied, and n = number of basidiospores measured from given number of specimens.

Crude DNA was extracted from basidiocarps of dry specimens using FH Plant DNA Kit (Beijing Demeter Biotech Co., Ltd., Beijing, China), and then directly used as template for subsequent PCR amplifications. The nLSU and ITS regions were amplified and sequenced using primer pairs LR0R and LR7^15^, and ITS1F^16^ and ITS4^17^, respectively. The PCR procedure was as follows: for nLSU region initial denaturation at 94 °C for 1 min, followed by 34 cycles at 94 °C for 30 s, 47.2 °C for 1 min and 72 °C for 1.5 min, and a final extension at 72 °C for 10 min, while for ITS region initial denaturation at 95 °C for 3 min, followed by 34 cycles at 94 °C for 40 s, 57.2 °C for 45 s and 72 °C for 1 min, and a final extension at 72 °C for 10 min. The PCR products were sequenced at the Beijing Genomics Institute, Beijing, China. The newly generated sequences were deposited in GenBank (https://www.ncbi.nlm.nih.gov/genbank/; Table 1).

The nLSU dataset was used to explore the phylogenetic position of *Basidioradulum*, and included sequences from all main lineages in *Hymenochaetales* and two species in *Polyporales* as ingroup taxa and *Amaurodon viridis* (Alb. & Schwein.) J. Schröt. in *Thelephorales* as an outgroup taxon. The ITS dataset was used to differentiate the phylogenetic relationships among specimens of *Basidioradulum*. The datasets were aligned using MAFFT 7.110^18^ under the g-ini-i option^19^, and the resulting alignments, after manual inspection, were deposited in TreeBASE (http://www.treebase.org; accession number 24505). jModelTest^20,21^ was used to estimate the best-fit evolutionary model of the two alignments. Maximum likelihood (ML) and Bayesian inference (BI) methods were utilized for phylogenetic analyses of the two alignments following the evolutionary models. The ML method was performed using raxmlGUI 1.2^22,23^ with the calculation of bootstrap (BS) replicates under the auto FC option^24^. The BI method was carried out using MrBayes 3.2^25^ with two independent runs, each including four chains of 10 million generations and starting from random trees. The first 25% of the sampled trees every 1000th generation was removed, and the other 75% trees were remained for constructing a 50% majority consensus tree and calculating Bayesian posterior probabilities (BPPs). Tracer 1.5 (http://tree.bio.ed.ac.uk/software/tracer/) was used to judge whether chains converged.

Besides phylogenetic analyses, the alignment resulted from the ITS dataset was also subjected to distance estimation using MEGA version 7^26^. The analysis preferences were as follows: p-distance substitution model including both transitions and transversions, uniform rates among sites, and pairwise deletion treatment.

## Data Availability

All data generated or analyzed during this study have been deposited in public databases as indicated.
